# Pulsed Raman Lasing in Diode-Pumped Multimode Graded-Index Fiber with Tuned Femtosecond-Laser-Inscribed Bragg Grating

**DOI:** 10.3390/mi16121315

**Published:** 2025-11-24

**Authors:** Alexey G. Kuznetsov, Vadim S. Terentyev, Alexander V. Dostovalov, Ilya N. Nemov, Sergey A. Babin

**Affiliations:** 1 Institute of Automation and Electrometry, 1 Ac. Koptyug Ave., Novosibirsk 630090, Russiababin@iae.nsk.su (S.A.B.); 2 Physics Department, Novosibirsk State University, 2 Pirogov Str., Novosibirsk 630090, Russia

**Keywords:** Raman laser, graded index fiber, tunable FBG, multimode fiber

## Abstract

Raman lasers based on multimode graded-index (GRIN) fibers directly pumped by laser diodes are the object of intensive research, promising to produce high-power, high-quality beams at new wavelengths. In this paper, we demonstrate a pulsed operation of such a laser based on a 1 km 100/140 GRIN fiber in which a resonator is formed by a pair of FBGs: a high-reflective broadband input FBG and a tunable narrowband output FBG inscribed by fs laser pulses in the fundamental mode area of the core. In addition to beam quality improvement, the output FBG modulated by a piezoelectric nanopositioner with a frequency of 20–180 Hz generates laser pulses with a duration of 23–1.2 ms, respectively. The maximum power reached is 22 watts, and the signal spectrum widens significantly with increased pumping (>2 nm from the central wavelength of 976 nm). The pulse generation method used in this work introduces wavelength chirp in the individual pulse, which can be used in sensing and other applications.

## 1. Introduction

Photonic integrated circuits (PICs) with hybrid integration of various chips into the same platform, using micro-optics and photonic wire bonding for interconnects and conventional optical fibers for external connections of the PICs, are one of the mainstream approaches in the development of new generation photonic devices for optical communications, sensing, optical computing, and information processing, etc.; see, e.g., [1,2,3,4]. Light sources are also becoming integrated either into PICs, in the case of low-power chip-scale lasers [5], or into external fiber schemes. Robust diode-pumped laser schemes with all-solid-state or all-fiber performance, where laser cavity optics and pump elements are integrated into a single unit, are one of the main directions of high-power laser development [6]. Hybrid schemes consisting of fiber and integrated optical elements, which are produced with the use of various micro-fabrication technologies, are also being actively developed recently [7].

One of the examples of such novel laser schemes is the Raman fiber laser (RFL) based on directly diode-pumped multimode graded-index (GRIN) fiber widely used in telecommunications; see [8,9,10] for a review. Due to the broadband Raman gain spectrum and in-fiber cavity consisting of fiber Bragg gratings (FBGs) micro-fabricated by point-by-point femtosecond (fs)-laser-inscription technology [10], such lasers allow for the high-efficiency generation of high-quality and high-power beams at new wavelengths that are not available from conventional fiber lasers [11], being also free from limiting effects such as mode instability and photo-darkening. The in-fiber FBGs also provide for the stable and robust all-fiber integrated performance of such lasers, as well as extreme beam quality improvement (to a nearly diffraction-limited one [12]), in addition to the Raman beam cleanup effect arising from the specifics of Raman gain in multimode GRIN fibers [13]. The technique of inscribing artificial random reflectors by fs laser pulses [14] also allows the control of the generated transverse mode composition in the multimode fiber. One can also obtain pulsed regimes (Q-switching and mode locking) in multimode RFLs with the use of an intra-cavity acousto-optic modulator, enabling the generation of nanosecond pulses; see [15] and the citation therein.

It is also possible to explore alternative opportunities of integrated design for multimode RFL using, instead of fs-inscribed FBGs or random reflective structures, an in-fiber output coupler based on an output dielectric mirror micro-fabricated on the central area of the GRIN fiber end face [16] by a micro-lithographic process similar to in [17,18,19]. After the femtosecond laser exposes the photoresist-coated fiber end face and the subsequent development process thus forms a hole, the output mirror with reflection R ~ 20% (one TiO_2_ layer) was deposited by magnetron sputtering on the central part of the GRIN fiber end face so that it gains transverse mode selective properties. Together with highly reflective (R > 90%) FBG, which is UV-inscribed in the core at the input end of the GRIN fiber where highly multimodal (M^2^ ~ 34) laser diode (LD)-pumped radiation is coupled to the fiber, it forms a linear cavity providing Raman lasing of a Stokes wave at 976 nm. In such a cavity, it appears possible to significantly reduce the threshold pump power (<80 W). The measured output beam quality factor amounts to a near diffraction-limited one (M^2^ < 2) near the threshold. With increased pumping to around 35 W output power at ~60% slope efficiency, a narrow spectrum has been demonstrated at the expense of a slight worsening of beam quality to M^2^ ~ 3, without any sign of mirror degradation at the achieved intensity of >30 MW/cm^2^. Due to broadband reflection of the mirror, electro-mechanical stretching of the FBG enables the tuning of the lasing wavelength from 972.7 to 979.1 nm (6.4 nm range) [20].

Here we study an alternative opportunity for micro-mechanical control of the operation regime for a multimode RFL with an in-fiber FBG cavity operating near 976 nm [12]. Implementing a fast electro-mechanical modulation of multimode FBG, we simply demonstrate a pulsed operation of the multimode RFL based on 1 km 100/140 GRIN fiber in which a resonator is formed by a pair of FBGs: a high-reflective broadband input FBG and a tunable narrowband output FBG inscribed by fs pulses in the fundamental mode area of the core. In addition to beam quality improvement, the output coupling FBG, modulated by a piezoelectric nanopositioner with a frequency of around 100 Hz, provides a simple way of generating laser pulses with a duration on the millisecond scale, depending on the modulation frequency. The maximum power reached is 22 watts, and the signal spectrum widens significantly with increased pumping (>2 nm from the central wavelength of 976 nm). Previously, experiments were conducted on pulse generation using the modulation of cavity FBGs so that their spectral overlap changed at modulation, but the published work concerned single-mode rare earth-doped fiber lasers [21,22]. So, in [21], a Q-switched erbium fiber laser was demonstrated with a pulse duration of 2.46 μs and a peak power of 2.1 mW. In [23], the authors also study a Q-switched Er-fiber laser, one of the FBGs of which is modulated by a piezoelectric tube with a frequency up to 18.5 kHz, which is the fundamental resonance of the tube. The frequency response of piezoelectric transducers is, in general, determined by the mechanical resonances of the device. In order to overcome the limitation of operation at a constant frequency, magnetostrictive rods were used in [24] as mechanical transducers to tune the Bragg wavelength of an FBG. The frequency response of the magnetostrictive transducer is rather flat, and a repetition rate from 0 to about 200 kHz was obtained. However, no works are known that investigate long (~1 km) multimode fiber Raman lasers with multimode pumping, using the technique of forming pulsed radiation by longitudinal modulation of the cavity FBG. These lasers have quite interesting specifics due to strong nonlinear effects and a complex mechanism of interaction of transverse modes compared to single-mode lasers. The laser we present combines the ability to tune the generation wavelength and, based on this, the formation of pulsed radiation with a power of tens of watts at wavelengths inaccessible to classic lasers on active fibers. Also, the pulse generation method used in the work introduces wavelength chirp in the individual pulse and spectral broadening, which is important for applications.

## 2. Materials and Methods

**Figure 1:**
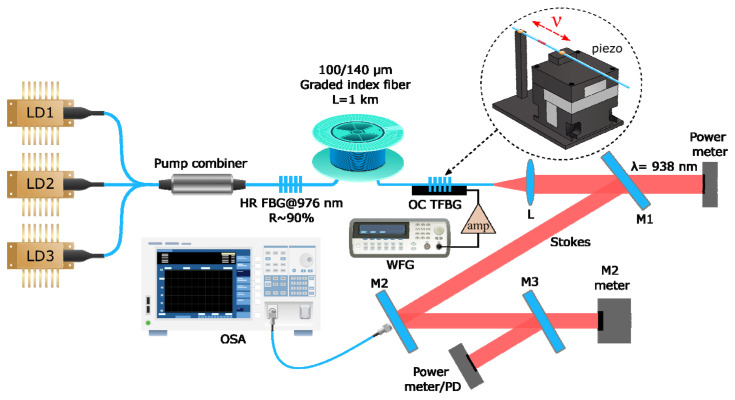
Scheme of a pulsed multimode Raman fiber laser: LD1–LD3—multimode laser diodes; HR FBG—high-reflective FBG inscribed using CW UV radiation; OC TFBG—output coupler tunable FBG inscribed by fs pulses; L—collimating lens; M1–M3—dichroic mirrors; OSA—optical spectrum analyzer; WFG—waveform generator with amplifier. The electro-mechanical module with a piezo positioner for FBG tuning with modulation frequency ν is shown in the inset.

**Figure 2:**
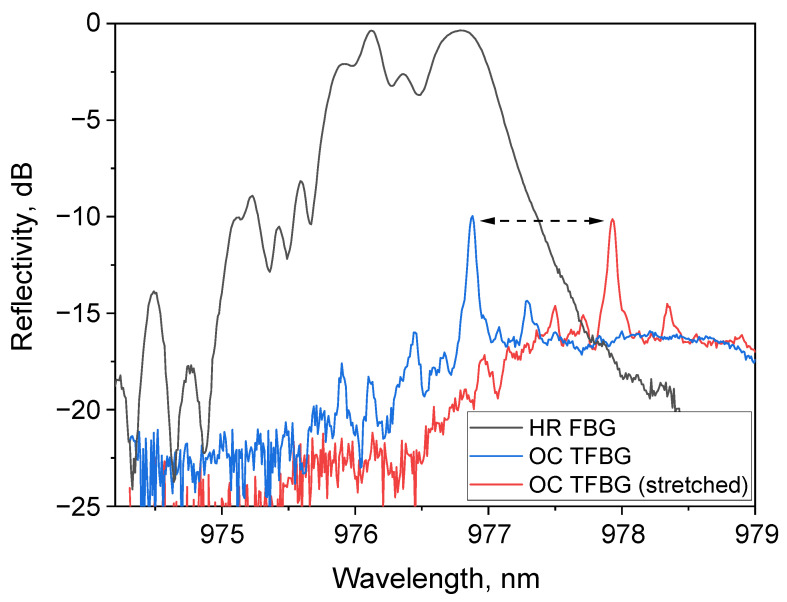
Reflectivity spectra of HR FBG and OC TFBG in free and stretched states.

The cavity scheme of the RFL under study is shown in Figure 1. The pump radiation from three high-power multimode laser diodes operating at a wavelength of ~940 nm is combined by a 3 × 1 multimode fiber pump combiner. Each multimode laser diode is pigtailed with a 105/125 µm multimode step-index fiber with numerical aperture NA = 0.22, has water cooling, and has a maximum output power of >100 W. The input ports of the fused combiner are also made of multimode fiber with a 105/125 step-index profile, and the output port is made of Draka 100/140 GRIN fiber with a 100 μm core and a numerical aperture of 0.29. It is spliced to the same 1 km long GRIN fiber with a 100 μm core, in which Raman amplification is provided by the laser pump. The linear resonator was formed by a CW UV-laser-inscribed high-reflective (HR) FBG (R ~ 90%) and an output coupling low-reflective (R ~ 10%) FBG (OC FBG) inscribed by fs laser pulses in the central cross-section of the fiber core. That is why the output FBG suppresses the generation of higher-order transverse modes, as dominant feedback is provided for the fundamental mode. The corresponding spectra of a broadband HR FBG and fs-inscribed OC FBG with a main narrow peak corresponding to the fundamental mode are shown in Figure 2. The spectra in the figure show that the central reflection wavelength of the HR FBG is 976.8 nm, and the full-width at half-maximum (FWHM) of the reflection spectrum is 0.54 nm. To the left of the main peak in the short-wavelength region, multiple peaks are present, corresponding to the Bragg resonances with higher-order transverse modes in the GRIN fiber. The reflection maximum of fs-inscribed OC FBG is located at a wavelength of 976.9 nm, and the FWHM bandwidth is only 0.08 nm. Since this FBG is inscribed in the central region of the core cross-section, the fundamental mode dominates in the reflection, while higher transverse mode resonances are suppressed. The output end of the fiber was angle-cleaved to minimize Fresnel back-reflection, which leads to the excitation of multiple transverse modes in the cavity that ultimately degrade the output beam.

The pump radiation transmitted through mirror M1 was measured by a power meter, and the Stokes radiation reflected from mirrors M1, M2, and M3 was measured by a second power meter. To detect the generated pulses over time, the power meter was replaced with a photodiode module. A portion of the residual radiation transmitted through M2 and M3 is used to measure the output spectrum and profile of the generated beam using a Yokogawa AQ6370 (Yokogawa Electric Corporation, Tokyo, Japan) optical spectrum analyzer (OSA) and a Thorlabs M^2^ measurement system (Thorlabs GmbH, Bergkirchen, Germany), respectively.

A special feature of the laser under study is that the output FBG has been made tunable (so it is named TFBG). To achieve this, it is attached to a piezo positioner, which stretches the FBG and thus shifts its reflection wavelength. We used the P-611.3 NanoCube Nanopositioner with a maximum axial displacement of 120 µm, an accuracy of 0.2 nm, and a resonant frequency of 220 Hz (unloaded). A sinusoidal electrical signal with a frequency of 20–180 Hz and an amplitude of 40 V was applied to the piezoelectric positioner, which corresponds to a maximum shift in the FBG reflection spectrum by ~1 nm due to the stretching. The plastic cladding was stripped from the fiber piece with the inscribed OC FBG; it was stretched between a fixed support and a piezoelectric positioner and glued at the corresponding two points with a UV light-curing glue.

As shown in Figure 2, the main reflectance peaks of the OC and HR FBGs coincide in the free state, but when the OC TFBG fs pulse is stretched, it shifts so that the reflectance level of the HR FBG at this spectral position falls to −17.2 dB. Thus, by making the OC FBG tunable (TFBG) and modulated, the FBG cavity periodically goes out of resonance, and it becomes possible to form a pulse train with a repetition rate corresponding to TFBG modulation frequency.

## 3. Results

**Figure 3:**
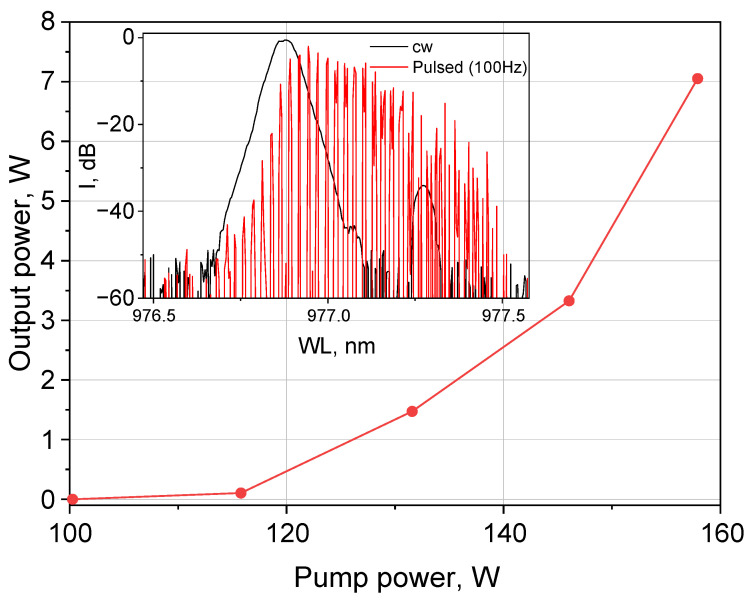
Measured output power of Stokes waves versus input pump power coupled to the GRIN fiber (100 Hz). Inset: laser spectrum in CW and pulsed (100 Hz) regime at 132 W pumping.

Figure 3 shows the measured average output power at the Stokes wavelength (~976 nm) of the stimulated Raman scattering (SRS) versus the LD pump power (~940 nm) at an FBG modulation frequency of 100 Hz. The lasing threshold was 115 W, and the output signal power at this repetition rate amounted to 7 W at the pump power of 158 W, which is lower than the output power in the CW regime (without FBG modulation). At that power, the laser spectrum in the pulsed regime significantly changes in comparison with the CW regime (see inset in Figure 3), which requires a comprehensive analysis of the temporal and spectral characteristics of the obtained pulses as a function of modulation frequency and pump power.

**Figure 4:**
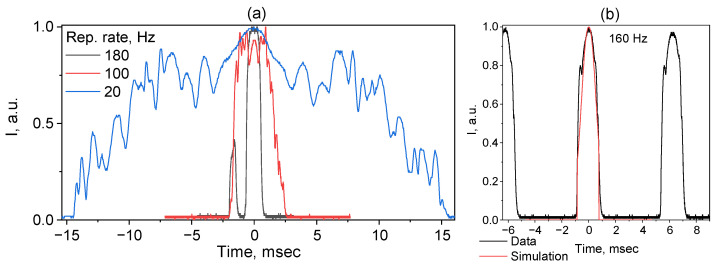
Pulse shapes at different repetition rates (158 W pump) (**a**) and typical pulse train at 160 Hz repetition rate with simulation result for pulse shape shown in red (**b**).

If we examine the temporal characteristics, we notice that both the pulse shape and its duration significantly depend on the frequency of the sinusoidal signal applied to the piezoelectric positioner. Thus, at 20 Hz modulation, the pulse has a maximum duration and its envelope is significantly jagged (Figure 4a), with some symmetry observed around the maximum. It can be assumed that the jaggedness is due to the Raman gain and the HR FBG reflection coefficient variations along its spectral profile within the TFBG scanning range. As the TFBG modulation frequency increases, the pulses shorten and smooth out. A significant shortening of each pulse to ~1 ms is evident, while its profile has acquired a smoother shape. With a further increase in the repetition rate to 180 Hz, which is close to the resonance of the piezo positioner, the pulse splits into two (with the second, weaker narrow peak lasting 0.3–0.4 ms and separated from the main peak by 1.7 ms). In Figure 4b, an optimal pulse train with a 160 Hz repetition rate is shown demonstrating smooth pulses with a duration of slightly longer than 1 ms and a period of about 6 ms, corresponding to the inverse modulation frequency. A simulation of pulse formation can be performed by recalculating the change in the reflection wavelength of the OC FBG over time and taking into account the reflection spectrum of the HR FBG (Figure 2). The simulation result for the pulse shape is also shown in Figure 4b and demonstrates good agreement with the experimental data.

**Figure 5:**
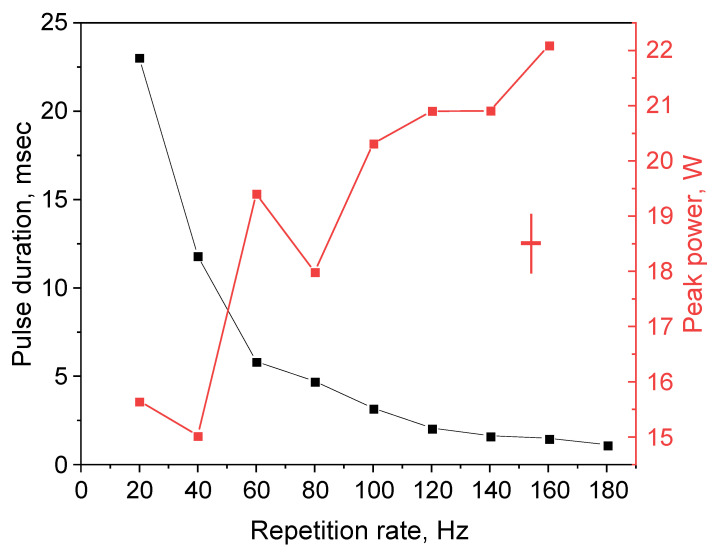
Pulse duration and peak power with repetition rate (pump 158 W). The cross indicates the characteristic errors of each point estimated from the pulse train data (Figure 4).

Figure 5 shows the dependence of the pulse duration on the repetition rate, as well as the corresponding peak power calculated from the average power and pulse duration. With increased repetition rate, the pulse duration decreases from ~23 ms to 1.2 ms, and the peak power increases from ~15 to 22 W when the repetition rate changes in the range 20–180 Hz.

**Figure 6:**
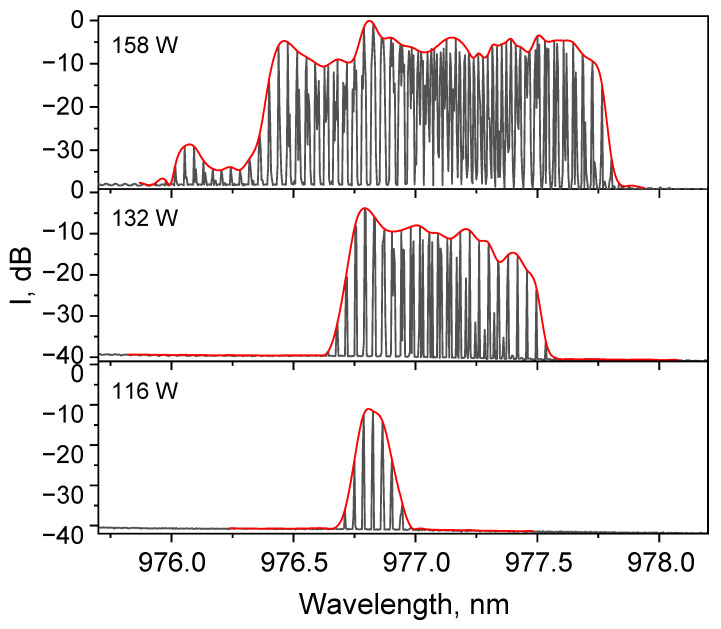
Output optical spectra with it’s envelop (red line) at different pump powers (repetition rate 120 Hz).

Figure 6 shows the lasing spectra at different pump powers. Since OSA scans quite slowly, the spectrum visually consists of peaks corresponding to the pulses, and the envelope should be taken to obtain a reliable lasing spectrum. Though there are no significant changes in the spectra at different modulation frequencies, the spectrum broadens sufficiently with increasing pump power. At low power the broadening occurs predominantly toward the long-wavelength region, and this corresponds to a modulation of the FBG reflectance spectrum between the extreme positions, as shown in Figure 2. Thus, the pulse acquires sequentially negative and positive wavelength chirp (at the TFBG movement from blue to red positions and back, see Figure 2). At the maximum pumping of 158 W, the lasing spectrum occupies the entire working range of the FBG modulation from 976.8 to almost 978 nm and extends even more in the short-wavelength part (to about 976 nm), so that the chirp rate value can be estimated as 0.7 nm/ms. With further increases in pump power, the gain at 978 nm (the reflection wavelength of the stretched OC TFBG) becomes higher than the loss, meaning lasing is not fully interrupted at the FBG stretching. In this case, the pulsed mode is replaced by a continuous one with intensity modulation. So, to resume pulse generation at high pump power, the OC TFBG wavelength modulation range must be increased to >1 nm.

**Figure 7:**
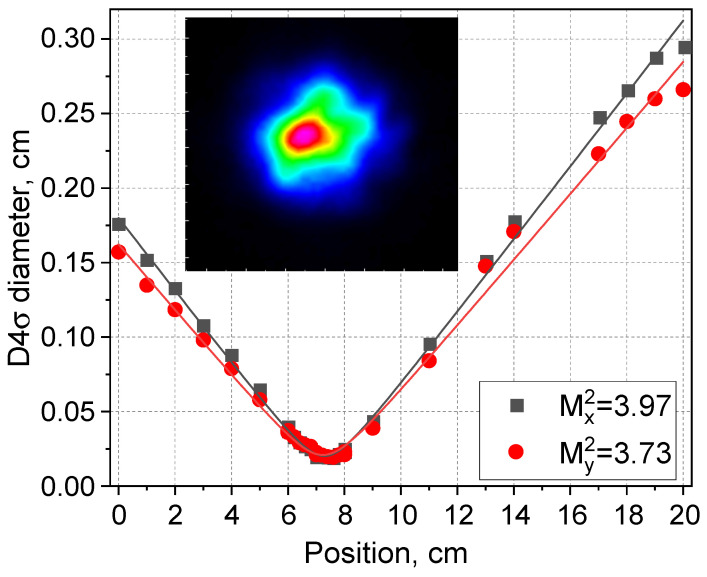
Measured beam quality parameter M^2^ and beam shape in the waist (inset) of pulsed (120 Hz, 158 W pump power) generation.

The average beam quality parameter at the 120 Hz repetition rate and 158 W pump was measured to be M^2^ ~ 3.8 (Figure 7), which is sufficiently better than the quality of the pump radiation beam (M^2^ ~ 34) due to the Raman beam-cleaning effect, similar to the CW operation of such lasers [21]. At the same time, the beam quality in the pulsed regime is slightly worse than that in the CW regime, as higher-order mode resonances of HR FBG in the short-wavelength range near 976 nm (see Figure 2) may be excited by the TFBG modulation, thus worsening beam quality when the short-wavelength domain is generated at maximum pump power of 158 W (see Figure 6). The reason for the beam degradation may be related, among other things, to mechanical factors of the stretching structure, such as inertial movement, when the FBG reflection line jumps further into the short-wave region corresponding to higher transverse mode resonances. To characterize this effect quantitatively, time-resolved beam quality measurements within a single pulse are required.

## 4. Discussion and Conclusions

Thus, a pulsed Raman laser with CW diode pumping of a multimode fiber, the cavity loss modulation in which is achieved by a tunable in-fiber FBG, has been demonstrated. Such a method is quite simple and does not disrupt all-fiber performance, which is applied to both Raman and multimode fiber lasers for the first time, to our knowledge.

It is shown that by modulating the narrowband output TFBG stretch with a piezo positioner, and thus changing its spectral position within the broad reflection spectrum of multimode HR FBG, it is possible in a multimode diode-pumped Raman fiber laser to generate pulse trains with a repetition rate corresponding to the modulation frequency. The duration of the pulses decreases with increasing modulation frequency, and in our case, they ranged from ~20 ms to ~1 ms at frequencies of 20 and 180 Hz, respectively. With increasing power, the width of the lasing spectrum of individual pulses increases, and the pulses acquire a chirp, the sign of which corresponds to the rise or fall of the pulse front. Since the Raman gain width is quite wide, the maximum width of the lasing spectrum is determined by the TFBG tuning range and the available pump power. The peak power of the pulses grows with increasing modulation frequency and reaches 22 W.

The effect of the peak power increase and pulse chirping in the obtained pulsed regime of the multimode RFL is important for applications, especially for the use of such a laser for temperature/strain sensor interrogation in various spectral domains—see, e.g., [25,26] and the citation therein. In the short-wavelength spectral domain around 1 µm, corresponding to the operation range of the developed laser, the losses are rather high, which requires high peak power, whereas pulse chirping of ~2 nm allows for monitoring temperature variations in an extended range. The chirping range may be further extended if a larger range of TFBG stretching at the piezoelectric modulation is applied, which also allows further increases in the pump power and lasing power as a result. Higher modulation frequencies are also important in this sense.

It is also interesting to tailor HR and OC FBG reflection spectrum shapes in order to optimize output beam quality and pulse train characteristics in terms of pulse duration, chirp, and their shape in temporal and spectral domains. By varying the initial (unstretched) position of the OC FBG reflection spectrum relative to the HR FBG spectral maximum, the chirp character can be changed. For example, if the initial position of the OC FBG reflection line is set to the left of the HR FBG peak, then two pulses with opposite chirps will be generated during one piezoelectric modulation period. Also of particular interest is the possibility of controlling and modulating the generated composition of the transverse modes when the low-reflectivity FBG resonates with the short-wavelength reflection peaks of the HR FBG, corresponding to higher transverse modes. Potentially, on the basis of such a laser, new interrogation techniques combining spectral and time domains may be developed and applied to distributed single and multimode fiber sensors based on FBG arrays.

## Data Availability

The original contributions presented in this study are included in the article. Further inquiries can be directed to the corresponding author.
